# Shedding light on maternal mental health in LMICs: a cornerstone of maternal and child health care

**DOI:** 10.1007/s44192-024-00111-3

**Published:** 2024-11-12

**Authors:** Alisha Handa, Abhay Gaidhane, Sonali Choudhari

**Affiliations:** Deparment of Community Medicine, Jawaharlal Nehru Medical College, Datta Meghe Institute of Higher Education and Research, Wardha, India

**Keywords:** Mental well-being, Psychological wellness, Postpartum depression, Mood disorders

## Abstract

Maternal and Child Health (MCH) programs have long been integral to global public health initiatives, aiming to safeguard the well-being of both mothers and their offspring. However, amidst the comprehensive approach to physical health, the mental well-being of mothers has often been overlooked, representing a critical gap in these programs. This paper examines the importance of addressing maternal mental health within the framework of MCH programs, highlighting its significance and the repercussions of its neglect. Despite its profound impact on maternal and child outcomes, issues such as postpartum depression, anxiety, and maternal stress are frequently disregarded in MCH interventions. This oversight not only undermines the holistic nature of maternal health but also perpetuates cycles of poor mental health within families and communities. Herewith, an effort was made to highlight the importance of maternal mental health and the need to focus and strengthen awareness about it through policy and programs.

## Introduction

‘A state of wellbeing in which a mother realizes her abilities, can cope with the normal stresses of life, can work productively and fruitfully, and is able to make a contribution to her community’ is how the WHO defines maternal mental health [1]. Around 10% of expecting mothers and 13% of those who have recently become mothers globally have mental health issues, with depression being the most common. In developing countries, the rates are 15.6% during pregnancy and 19.8% after childbirth. These are noticeably higher [2]. Significant problems associated with women's pregnancies and postpartum periods include perinatal mental illnesses [3]. Poor mental health among women in their perinatal era is a common occurrence. These problems might range from modest psychological distress that passes quickly to persistent, progressive, and profoundly disabling conditions.

In the medical community, mental health illnesses are commonly referred to as "disorders" because they are characterized by clinically significant changes in thoughts, perceptions, emotions, or behaviour. They are also frequently linked to distress, functioning impairment, or the risk of self-harm [4, 5].

Maternal and child health (MCH) initiatives focus traditionally on physical health, and mental health is quite often overlooked. The most prevalent mental health condition among mothers is depression, which is followed by bipolar illness, postpartum psychosis, anxiety disorders (such as generalized anxiety disorder, panic disorder, obsessive–compulsive disorder, and birth-related PTSD), and depression. Maternal mental health issues are seen as a significant global public health concern. While maternal mortality remains the primary determinant of maternal health, the World Health Organization is proposing metrics related to healthy life expectancy and universal health coverage. This suggests that mental health issues will receive more attention in the integrated delivery of maternity and child health services [2].

In rural India, the circumstances for women who struggle with mental health issues during and after pregnancy are dire. In rural India, perinatal mental health issues are highly prevalent and frequently go undiagnosed and untreated, but empirical research on the subject is lacking [6]. In India, according to specific research conducted in low- and middle-income countries (LMICs), the estimated prevalence is significantly greater, at 15.6% prenatal and 19.8% postnatal [7]. Lower socioeconomic levels, inadequate education, unemployment, and strained relationships with in-laws are associated with mental health issues in perinatal women throughout India [8]. Particular sociocultural elements like gender related vulnerability to psychopathology and to psychosocial stressors, being single, childhood adversities including physical, emotional and sexual abuse, dowry disputes have an impact on pregnant women's suicide risk in India [9].

When maternity services were enhanced and training was provided for rural midwives and birth attendants in the early 1900s, MCH was first implemented. The Maternal and Child Welfare Bureau of the Indian Red Cross Society oversaw the voluntary effort known as MCH. In 1931, Madras State became the first to create a distinct Maternal Welfare division inside the Office of the Director of Health Services. The Bhore Committee suggested in 1946 that MCH be integrated into General Health Services; however, this recommendation was only put into practice after 1955. Before 1953, midwives and maternity homes provided MCH, which was dispersed unevenly [10]. The fundamental principles of fairness, universal care, entitlement, and accountability serve as a roadmap for the programmes and initiatives created by different divisions to provide a "continuum of care" that equally prioritizes different life stages [11]. The objectives of maternal mental health care are the reduction of maternal, perinatal, infant and childhood mortality and morbidity; promotion of reproductive health; promotion of the physical and psychological development of the child and adolescent within the family and the ultimate objective of MCH services is lifelong health.

## The importance of maternal mental health

1. *Maternal well-being*: Hormonal changes, lack of sleep, and adjusting to a new role accompany the journey to parenthood may have an adverse effect on a woman's mental health [12]. Hormonal fluctuations during pregnancy and childbirth are profound. Particularly noteworthy is the sudden drop in progesterone and oestrogen levels following birth. These hormone shifts have the potential to significantly affect the brain's processes for regulating mood. Because of the complex interactions that exist between these changes in hormone levels and the activity of neurotransmitters like serotonin, oxytocin, dopamine, mothers may be more vulnerable to mood problems after giving birth [13]. Maternal well-being is important for the mother, child and the family as a whole.
2. *Child development*: It has been noted that children born to postpartum depressive mothers have dysregulated attention and arousal patterns. Children raised by depressed mothers are less likely to get contingent stimulation, which interferes with their ability to complete non-social learning tasks [14]. Mothers who are depressed may have lower rates of setting and keeping boundaries with their children [15]. Children of depressive mothers show less developed expressions of age-appropriate autonomy and appear more passively non-compliant [16].
3. *Bonding and attachment*: There are intrinsic physiological, psychological, and emotional relationship between mothers and their children. Maternal-infant bonding is an expression used to describe the attachment between a mother and her child. Some mothers may find it challenging to provide and focus the necessary warmth and affection for their child; this condition is known as impaired or disordered maternal-infant bonding [17] and is characterized by feelings of anger, rejection, or irritability towards the child [18]. The attachment of the child to the mother is influenced more in cases when depression coexists with another mental illness or it manifests before the prenatal period.
4. *Family dynamics*: During the postpartum phase, partners become essential support providers, providing comprehension, aid, and emotional bonding. However, the difficulties brought on by a mother's mental health problem might cause tensions that spread across the family. As they try to figure out how to give the required care and support while managing their emotions, the partner's own emotional health may suffer. Extended family members are also crucial because of their compassion and participation, which can significantly lessen the mother's burden. Their support and involvement can facilitate the development of a more favourable atmosphere for the mother's recovery and the family's welfare [19].
5. *Workforce productivity*: Parenting can be discouraged by the notion that having a child can impede one's ability to pursue paid employment. Maternal mental illnesses negatively impact a woman's overall quality of life, including her ability to function at work [15]. Recent mothers may view this as a stressful situation despite the latent psychosocial benefits of employment and actual evidence that women respond well to returning to work after childbirth as they have a healthy and motivated employee support which are fundamental at the organizational level and for the socioeconomic wellbeing of any country. This does vary depending on the nature of work the mother is involved in [20].

## Barriers to addressing maternal mental health

Maternal mental health receives inadequate attention globally from the healthcare delivery system despite its significance. Several barriers contribute to this oversight.

1. *Stigma-Public stigma/experienced stigma*: Due to their mental health condition, pregnant women are often subject to prejudice or discrimination [21]. The potential impact of this could be self-isolation and lack of health-seeking behaviour. This would, in turn, increase the psychiatric morbidities and also impact physical health outcomes if women don’t seek help generally.
  Mothers with mental illnesses experience self-stigma or internalized stigma when they project others' critical or unfavourable attitudes onto themselves [22].
  *Associated stigma*: When a pregnant woman with a mental condition is linked to her family, that family faces stigma.
  *Structural stigma*: Infant care and community involvement may be denied to pregnant women who have mental illnesses.
  *Label avoidance*: Because getting mental health treatment will automatically classify a pregnant woman as having a mental disorder, she may choose not to seek it.

In our experience of the (LMICs)it shows that stigmatization of perinatal women with mental illness is common when it comes to seeking treatment, refusing treatment, taking medication, and having a spouse or family member (such as a mother) with a mental illness. This frequently leads to family upheaval (divorce, for example), financial strain on the family, and social disengagement of pregnant women with mental health disorders. These detrimental effects prolong the stigmatization loop and aggravate mental health problems. The lack of help seeking behaviour for prevalent prenatal mental issues is caused by stigma. This is associated with poor child outcomes and raises the risk of substance abuse and suicide among pregnant mothers [23].

2. *Lack of screening* Unhealthy community customs make it more difficult to seek medical attention. The custom in the society of concealing women who show symptoms of mental illness is another seen in society, referring to LMICs broadly. According to respondents of the study done in Uganda, stigma and unfavourable societal opinions of mental illness are most likely the driving forces for this practice. There is a lack of awareness regarding mental health in the community. The stigma associated with mental health in the community prevents many women from accessing the necessary medical care, which exacerbates their suffering [24] which in turn leads to women not opting for screening for their mental illness.
3. *Limited resources* There is a shortage of medications and mental health professionals to treat prevalent maternal mental health diseases. Among the difficulties encountered, a shortage of mental health professionals and medications for the most pervasive MMH problems is seen. It is challenging for non-specialized care providers to consult with psychiatric nurses, clinical officers, and doctors due to the shortage of these professionals. In comparison to the number of mental health professionals available, the number of patients is far too high [24].
4. *Fragmented care* MCH services are often approached incoherently in many healthcare systems due to the division of mental health services from obstetric and paediatric care. The absence of integration among various services may lead to omissions of chances for early intervention and support, communication breakdowns amongst providers, and gaps in care coordination. As a result, mothers could slip through the gaps in the healthcare system, worsening their mental health issues and endangering her and her child’s’' well-being [25].

## Integrating maternal mental health into the MCH perspective

To address these challenges, a multifaceted approach is needed to integrate maternal mental health into the MCH perspectives effectively:

1. *Awareness and Education* One of the main goals of India's National Mental Health Program (NMHP) is to de-stigmatize mental illness through informational, educational, and communicative initiatives. It collaborates with the district mental health programme, a state administrative division that carries out the NMHP [26]. The rights of people with mental illness should be upheld; stigma should be lessened, community involvement should be encouraged, mental health care services should be more widely accessible, staff should be empowered and motivated in the workplace, mental health services should be delivered with better infrastructure, knowledge and evidence should be produced for service delivery, and governance, administrative, and accountability mechanisms should be established [27]. Education for women, families, communities, and healthcare professionals can foster de-stigmatisation and understanding. Awareness and education of perinatal mental health should be given to mothers and they should be made aware of the signs and symptoms of mental health problems. The third Sustainable Development Goal (SDG) aims to "Ensure healthy lives and promote well-being for all at all ages." This objective covers a broad spectrum of health-related goals, such as lowering the burden of non-communicable diseases, increasing mental health and wellbeing, and providing universal access to healthcare. A major factor in tackling mental health in India is the National Mental Health Programme (NMHP), which is in line with a number of SDG Goal 3 aims.
  The two are connected as follows:
  1. SDG Target 3.4: Advance Mental Health and Well-Being
    By fostering mental health and well-being, target 3.4 expressly asks for a one-third decrease in the premature death rate from non-communicable diseases, which also includes mental health disorders.
  2. By emphasising early diagnosis, treatment, and rehabilitation of those suffering from mental health issues, India's National Mental Health Programme (NMHP) directly helps to accomplish its goal of providing mental health services to all segments of the community [28].
2. *Routine screening* It is justified to screen for anxiety and depression during pregnancy to improve the health of both the mother and her unborn child. It has been demonstrated that taking part in perinatal depression screening programmes enhances the identification of women who are at risk, referral uptake, and service engagement, all of which have a favourable effect on mental health outcomes [29]. It is advised in high-income nations like the United States of America, Australia, and the United Kingdom to conduct routine, standardized mental health screenings throughout pregnancy and this approach should be implemented in India as well. However, there is a frequent evidence-practice gap in this regard, with screening being inadequately adopted because women and health professionals lack information and assistance [30]. Screening tools like the Edinburg postnatal depression scale (EPDS), generalized anxiety disorder, postpartum depression screening scale (PDSS), beck depression inventory (BDI) can be used for screening that should be culturally sensitive and integrated into the existing healthcare workflow.
3. *Collaborative care models* Postpartum mood disorders are treated using a multidisciplinary approach in collaborative care settings. To address the needs of women who have or are at risk of these disorders, these models often involve collaboration between healthcare professionals, mental health specialists, and social support agencies. Individualized treatment planning, thorough assessment, and frequent follow-up are all stressed in collaborative care approaches. Studies have demonstrated beneficial effects of these models, such as decreased intensity of symptoms, higher adherence to therapy, better quality of life, and improved mental health of mothers [31].
4. *Capacity building* Capacity building can be done by developing skills in screening, assessment and evidence-based interventions like early diagnosis through established screening tools, proper treatment through protocols that are well-defined for healthcare practitioners, and, in extreme circumstances through a risk based approach, using pharmacological therapy through inexpensive psychiatric medication that weighs the dangers. Psychoeducational therapies can be used that blend guidance with emotional support. Improving a mother's attentiveness and awareness of her infant's growing needs for comfort, stimulation, and engagement can lead to better mother–child relationships. Enhancement of mother-father work sharing and parenting, reduction of partner and family violence, and promotion of gender equality can all lead to better partner relationships. Brief psychological therapies that are solution-focused and culturally sensitive, increase in women's social support and enhancement of girls' and women's access to education and career training are some ways of capacity building [1].
5. *Policy and Advocacy* Integrating maternal mental health into primary care MCH programs by incorporating early detection, prevention, and treatment of maternal mental health issues like depression, primary care mental health initiatives may be able to close the treatment gap. Intersectoral cooperation and health system strategies that emphasize prevention and treatment across the life course and the use evidence-based interventions are helpful for such a kind of integration. Studies carried out in LMICs demonstrate the efficacy of psychosocial, educational, and supportive treatments in enhancing the mental health of mothers, even when provided by non-mental health specialists as opposed to specialists. These non-specialist employees can be crucial in helping individuals who most need primary mental health services and are not able to receive them [32].

## Limitations

Maternal mental health issues can significantly vary across different LMICs due to diversity in cultural, regional and socio-economic factors. Many LMICs have inadequate infrastructure for providing maternal mental health care services, which may affect the ability to diagnose, treat, or even study maternal mental health issues comprehensively. Most mental health screening tools are curated in the developed countries and may not be fully validated or adapted in LMICs, which could lead to inaccuracies in diagnosis or reporting.

## Conclusion

Addressing maternal mental health is crucial for the well-being of both mothers and her children. Early identification and intervention can help prevent long-term consequences and improve outcomes for both mother and child. Healthcare providers play a critical role in screening for maternal mental health issues during prenatal and postnatal appointments, providing education and support, and connecting women with appropriate resources and treatment options. Family and friends can also offer valuable support by listening, offering practical help, and encouraging mothers to seek help. Integrating maternal mental health into MCH programs is not merely an option but a necessity for promoting the holistic well-being of mothers, children, and families. Throughout this discourse, we've underscored the importance of recognizing and addressing maternal mental health as an integral component of MCH initiatives, emphasizing its profound impact on maternal, child, and familial outcomes. From the challenges of stigma and limited resources to the imperative for policy reform and collaborative care models, addressing maternal mental health within MCH programs requires a concerted effort across multiple fronts. By advocating for policy changes, investing in education and training, implementing routine screening protocols, fostering collaboration between healthcare providers, engaging communities, and supporting research endeavours, we can pave the way for a more inclusive and practical approach to maternal mental health care.

## Data Availability

No datasets were generated or analysed during the current study.

## Code availability

Software application or custom code-NA.
